# Mannose Binding Lectin Is Required for Alphavirus-Induced Arthritis/Myositis

**DOI:** 10.1371/journal.ppat.1002586

**Published:** 2012-03-22

**Authors:** Bronwyn M. Gunn, Thomas E. Morrison, Alan C. Whitmore, Lance K. Blevins, Linda Hueston, Robert J. Fraser, Lara J. Herrero, Ruben Ramirez, Paul N. Smith, Suresh Mahalingam, Mark T. Heise

**Affiliations:** 1 Department of Microbiology and Immunology, University of North Carolina at Chapel Hill, Chapel Hill, North Carolina, United States of America; 2 Carolina Vaccine Institute, University of North Carolina at Chapel Hill, Chapel Hill, North Carolina, United States of America; 3 Department of Microbiology, University of Colorado School of Medicine, Aurora, Colorado, United States of America; 4 Arbovirus Emerging Disease Unit, CIDMLS-ICPMR, Westmead Hospital, Westmead, Australia; 5 Department of Medicine, Royal Melbourne Hospital, University of Melbourne, Melbourne, Australia; 6 Virus and Inflammation Research Group, University of Canberra, Canberra, Australia; 7 Emerging Viruses and Inflammation Research Group, Institute for Glycomics, Griffith University, Gold Coast, Australia; 8 The Australian National University, Canberra, Australia; 9 Department of Genetics, University of North Carolina at Chapel Hill, Chapel Hill, North Carolina, United States of America; Washington University School of Medicine, United States of America

## Abstract

Mosquito-borne alphaviruses such as chikungunya virus and Ross River virus (RRV) are emerging pathogens capable of causing large-scale epidemics of virus-induced arthritis and myositis. The pathology of RRV-induced disease in both humans and mice is associated with induction of the host inflammatory response within the muscle and joints, and prior studies have demonstrated that the host complement system contributes to development of disease. In this study, we have used a mouse model of RRV-induced disease to identify and characterize which complement activation pathways mediate disease progression after infection, and we have identified the mannose binding lectin (MBL) pathway, but not the classical or alternative complement activation pathways, as essential for development of RRV-induced disease. MBL deposition was enhanced in RRV infected muscle tissue from wild type mice and RRV infected MBL deficient mice exhibited reduced disease, tissue damage, and complement deposition compared to wild-type mice. In contrast, mice deficient for key components of the classical or alternative complement activation pathways still developed severe RRV-induced disease. Further characterization of MBL deficient mice demonstrated that similar to C3^−/−^ mice, viral replication and inflammatory cell recruitment were equivalent to wild type animals, suggesting that RRV-mediated induction of complement dependent immune pathology is largely MBL dependent. Consistent with these findings, human patients diagnosed with RRV disease had elevated serum MBL levels compared to healthy controls, and MBL levels in the serum and synovial fluid correlated with severity of disease. These findings demonstrate a role for MBL in promoting RRV-induced disease in both mice and humans and suggest that the MBL pathway of complement activation may be an effective target for therapeutic intervention for humans suffering from RRV-induced arthritis and myositis.

## Author Summary

Arthritogenic alphaviruses such as Ross River virus (RRV) and chikungunya virus are transmitted to humans by mosquitoes and cause epidemics of debilitating infectious arthritis and myositis in various areas around the world. Studies in humans and mice indicate that the host inflammatory response is critical for development of RRV-induced arthritis and myositis, and the host complement system, a component of the host inflammatory response, plays an essential role in the development of RRV-induced disease through activation of complement receptor 3 (CR3)-bearing inflammatory cells. Of the three main complement activation pathways, only the lectin pathway activated by mannose binding lectin (MBL) was essential for RRV-induced complement activation, tissue destruction, and disease. Furthermore, we found that levels of MBL were elevated in human patients suffering from RRV-induced polyarthritis and MBL levels correlated with disease severity. Taken together, our data implicates a role for MBL in mediating RRV-induced disease in both humans and mice, and suggests that therapeutic targeting of MBL may be an effective strategy for disease treatment in humans.

## Introduction

Arthritogenic alphaviruses, such as Ross River virus (RRV) and chikungunya virus (CHIKV), are mosquito-borne viruses that cause severe polyarthritis and myositis in humans. RRV causes annual disease outbreaks in Australia and has caused sporadic epidemics of debilitating polyarthritis, including one outbreak involving over 60,000 people in Oceania [1]. RRV is transmitted to humans primarily by the *Aedes* and *Culex* species of mosquitoes that generally populate marsh areas, and CHIKV transmission has been traditionally mediated by the urban *Aedes aegypti*, though the virus has recently adapted for efficient transmission by the widely distributed *Aedes albopictus* species [2], leading to an increased risk for CHIKV spread into new areas, as illustrated by recent outbreaks in Italy and southern France [3]. The expansion of CHIKV into an additional mosquito vector and the subsequent epidemic has highlighted the ability of the arthritic alphaviruses to move into new geographic areas and cause large-scale outbreaks of acute and persistent arthralgia and myalgia in humans.

RRV-induced arthritic disease presents predominantly as painful stiffness, inflammation, and swelling in peripheral joints that can last months after initial infection and the host inflammatory response is thought to play a major role in disease pathogenesis. Inflammatory monocytes constitute the bulk of leukocytes isolated in synovial aspirates from RRV-infected patients [4], [5], and macrophage-cytotoxic drugs have been shown to drastically reduce disease progression and severity in mice [6], [7]. In addition, mice lacking C3, the central complement factor that is essential for complement activation, exhibit reduced RRV-induced disease and tissue destruction [8], implicating a role for complement in development of the disease. Consistent with studies in mice, synovial aspirates from patients with RRV-induced arthritis have been shown to contain increased levels of the C3 cleavage product C3a [8]. Although macrophage recruitment to infected tissues is markedly increased after RRV infection, the role played by complement is independent of inflammatory cell recruitment. Rather, tissue destruction and disease progression requires complement receptor 3 (CR3), suggesting that complement interactions with CR3 on inflammatory cells promote tissue destruction in RRV-infected tissues [9]. However, it is currently unclear how the complement system is activated following RRV infection.

There are three main activation pathways of the complement cascade; the classical, alternative, and lectin dependent pathways, that all converge on factor C3 and lead to activation of complement effector functions (reviewed in [10]). The classical pathway is initiated by C1q interactions with antigen-bound complexes of IgG and IgM, and the proteases C1r and C1s cleave C4 and C2 to generate the C3 convertase C4b2b. Binding of factor B (fB) and spontaneously hydrolyzed C3 initiates the alternative pathway, and fB binding to C3b leads to formation of the alternative C3 convertase C3bBb that can amplify complement activation. In the lectin pathway, mannose binding lectin (MBL) or the ficolins bind to carbohydrate moieties on foreign bodies, such as viruses, or to host cells and apoptotic cells, and the MBL-associated serine proteases (MASPs) cleave C4 and C2 to form the C3 convertase C4b2b. Cleavage and processing of C3 by the C3 convertases produce several C3-derived components that are potent activators of the immune system. One such component is iC3b, which is a ligand for several complement receptors, such as CR3. Binding of iC3b to CR3 on cells such as monocytes/macrophages, neutrophils, and NK cells, results in activation of these cells, leading to enhanced phagocytosis and cytotoxic activity against iC3b-opsonized cells [10].

MBL is a soluble C-type lectin that can initiate the complement cascade through binding of the carbohydrate recognition domains (CRD) to cell-surface sugars expressed on bacteria and viruses and some endogenous host ligands (reviewed in [11]). The complement system is notorious for having both a protective and pathologic role and frequently leads to additional tissue injury and damage once activated. Similarly, MBL appears to be able to have the ability to protect as well as harm the host cells. In the context of sterile inflammatory diseases such as myocardial and gastrointestinal ischemic reperfusion injury, MBL and the lectin pathway mediate development of disease through complement-mediated regulation of pro-inflammatory cytokines and inflammation, leading to exacerbated pathology and tissue injury [12]–[15]. In contrast to the pathologic role of MBL in sterile inflammatory diseases, MBL is thought to primarily play a protective role in response to infectious pathogens. MBL has been shown to be essential for host protection from many different viral and bacterial infections either through direct binding to pathogens or by limiting spread through complement effector functions. MBL has been shown to bind directly to many different viruses, including human immunodeficiency virus (HIV), Ebola virus, and arboviruses such as dengue virus and West Nile virus (WNV), and MBL can either directly neutralize these viruses through activation of complement or interfere with their binding to host cells (reviewed in [16]). Furthermore, studies using mice deficient in both MBL genes (MBL-A^−/−^ and MBL-C^−/−^; MBL-DKO) have revealed that MBL can have a protective role during WNV and herpes simplex virus infections [17]–[19]. Though MBL neutralizes flaviviruses, such as WNV and dengue virus [18], and plays a protective role during WNV infection [19], these same studies found no detectable interactions between MBL and alphaviruses, suggesting that MBL does not play a protective role during alphavirus infection. However, the role of MBL role in the pathogenesis of alphavirus-induced arthritis/myositis has not been evaluated.

The goal of this study was to further assess the role of the host complement system in the pathogenesis of alphavirus-induced inflammatory disease and to determine which complement activation pathways are required for virus-induced disease. In a mouse model of RRV-induced arthritis and myositis, mice deficient in either the classical or alternative pathways developed severe disease, while mice deficient in both genes of MBL (MBL-DKO) were resistant to disease, suggesting that MBL plays a major role in RRV-induced disease. Similar to previous findings with C3^−/−^ mice [8], RRV-infected MBL-DKO mice had similar levels of viral burden and inflammation compared to wild-type (WT) but exhibited significantly less complement deposition, tissue damage, and disease. Further analysis found that MBL levels are enhanced in RRV infected tissues and that MBL binds to RRV infected cells, suggesting that RRV infection leads to MBL deposition and subsequent complement activation. Importantly, studies in human patients suffering from RRV-induced disease found that levels of MBL were elevated in the serum of RRV-infected patients compared to healthy controls. In addition, serum and synovial fluid MBL levels correlated with the severity of RRV disease, while no differences were observed in classical or alternative pathway activation, suggesting that MBL contributes to RRV-induced disease in human populations.

## Results

### The MBL pathway is essential for RRV-induced disease and inflammatory tissue destruction

**Figure 1:**
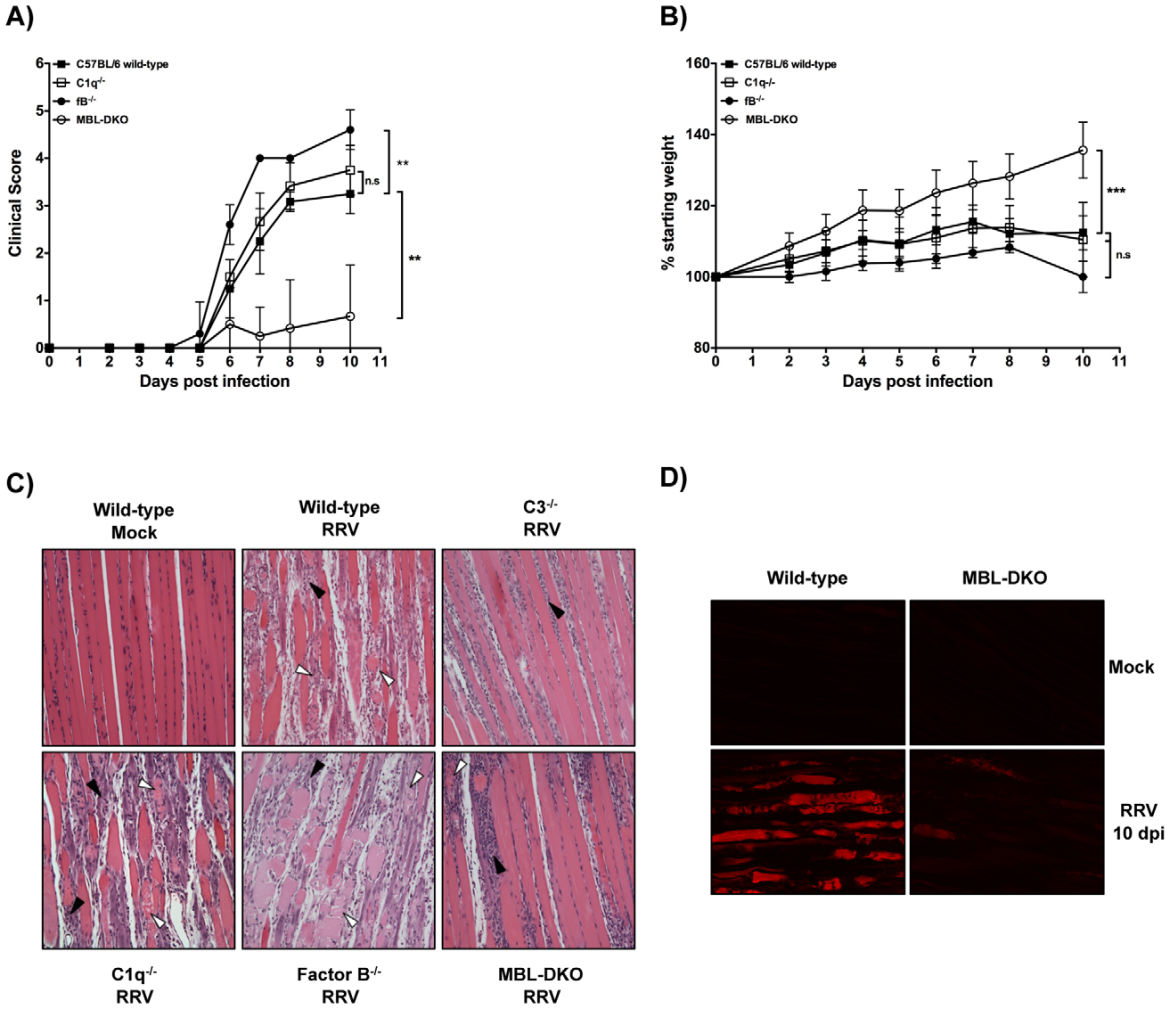
MBL is required for development of severe RRV-induced disease and tissue damage. (A–B) Twenty-four day old WT C57BL/6 (solid square, n = 6), C1q^−/−^ (open square, n = 6), fB^−/−^ (solid circle, n = 5), or MBL-DKO (open circle, n = 6) mice were infected with RRV, scored for hind limb function based on a scale described in the Materials and Methods, and assessed for weight loss. Each data point represents the arithmetic mean ±SD and is representative of at least three independent experiments. We performed a Mann-Whitney analysis with multiple comparison corrections (p<0.01 was considered significant) on clinical scores at 10 dpi and a one-way ANOVA analysis with Bonferroni's correction on percent of starting weight at 10 dpi (p<0.05 was considered significant) to determine significance between the various knock-out lines compared to WT **p<0.01; *** p<0.001; n.s. not significant. (C) Tissue pathology and inflammation was examined at 10 dpi by H&E staining of paraffin embedded sections of quadriceps muscle. A representative section from each knockout strain is shown. A section from RRV-infected C3^−/−^ is shown for comparison. Solid arrowheads point to areas of inflammation; open arrowheads indicate tissue damage (D) To assess damage within the muscle, mock or RRV-infected WT or MBL-DKO mice were injected with EBD at 10 dpi, and frozen sections were generated. EBD positive muscle fibers were identified by fluorescence microscopy. Representative sections of mock- and RRV-infected mice are shown and are representative of two independent experiments.

Complement activation products are elevated in the synovial fluid of persons suffering from RRV-induced arthritis and complement activation is required for virus-induced arthritis/myositis in a mouse model [8], [9], [20]. Although other alphaviruses, such as the neurovirulent Sindbis virus, have been shown to activate complement via both the classical and alternative pathways [21], the pathway(s) leading to complement activation by arthritic alphaviruses is currently unknown. Therefore, mice deficient in key components of the classical (C1q^−/−^), alternative (factor B, fB^−/−^), or lectin (MBL-A/C^−/−^, MBL-DKO) pathways were assessed for their susceptibility to RRV-induced disease. C1q^−/−^, fB^−/−^, MBL-DKO, or WT C57BL/6 mice were inoculated with RRV in the footpad and assessed for weight loss and scored for hind limb function as previously described [20]. RRV-infected WT mice showed signs of hind-limb weakness by 5 days post infection (dpi) and developed severe hind-limb weakness by 7 dpi through 10 dpi (Figure 1A). RRV causes disease independently of B cells and antibody [20], suggesting that the classical pathway does not contribute to disease during RRV infection. Consistent with this, C1q^−/−^ mice exhibited severe disease signs and hind-limb weakness similar to WT animals (Figure 1A). Likewise, fB^−/−^ mice developed severe RRV-induced disease (Figure 1A), demonstrating that the alternative pathway of complement activation is not required for RRV-induced disease, though it is important to note that RRV-infected fB^−/−^ mice tended to develop more severe disease compared to WT mice, suggesting that the alternative pathway is activated and may actually play a protective role during RRV infection. In contrast to C1q^−/−^ and fB^−/−^ mice, MBL-DKO mice infected with RRV developed mild hind-limb weakness and exhibited reduced weight loss compared to WT mice (Figure 1, A and B). Mock-infected WT and MBL-DKO mice did not differ in weight gain (Figure S1) and showed no signs of disease throughout the course of infection (data not shown). While we cannot rule out a minor contribution of the classical and alternative activation pathways or activation of the lectin pathway through ficolins in development of RRV disease, this data demonstrates that the lectin pathway initiated by MBL plays an essential role in driving RRV-induced disease.

### MBL contributes to damage within quadriceps muscle

Following infection of WT C57BL/6 mice, RRV replicates to high levels within both the joints and skeletal muscle and elicits an inflammatory infiltrate into these tissues [20]. Following the onset of inflammatory cell infiltration, wild type mice develop severe destructive myositis, which is a major aspect of virus-induced disease in this mouse model, and we have previously shown that muscle cell killing and disease is dependent upon both C3 activation and CR3 [8], [9]. Therefore, to confirm the role of MBL in driving RRV-induced disease, inflammatory pathology was assessed within the quadriceps muscles of RRV-infected fB^−/−^, C1q^−/−^, MBL-DKO, or WT mice by H&E staining of paraffin embedded sections. At 10 dpi, a time point of peak RRV disease, we observed similar inflammation and tissue pathology in fB^−/−^, C1q^−/−^, and WT mice (Figure 1C). Inflammatory cells are present in the quadriceps muscles of infected mice from all strains, as indicated by the solid arrowheads. We observed tissue damage in the quadriceps muscles in fB^−/−^, C1q^−/−^, and WT mice as evidenced by the degeneration of the fibrous architecture of the skeletal muscle (marked by open arrowheads). In contrast, RRV infected MBL-DKO mice maintained the architecture of the skeletal muscle with very little tissue damage, despite the presence of inflammatory cells (Figure 1C), which was strikingly similar to previous results demonstrating an essential role for C3 in RRV-induced disease [8]. To confirm that MBL-DKO mice have decreased tissue damage following RRV infection compared to WT mice, we used Evans Blue dye (EBD) uptake to detect areas of damage. Consistent with the clinical scores and histological analyses at 10 dpi, RRV-infected WT mice had abundant EBD positive muscle fibers within the quadriceps muscle whereas EBD positive cells were rare in RRV-infected MBL-DKO mice (Figure 1D), further demonstrating that MBL is required for the induction of tissue damage during RRV-induced disease.

### RRV infection induces MBL deposition onto tissues and cells

**Figure 2:**
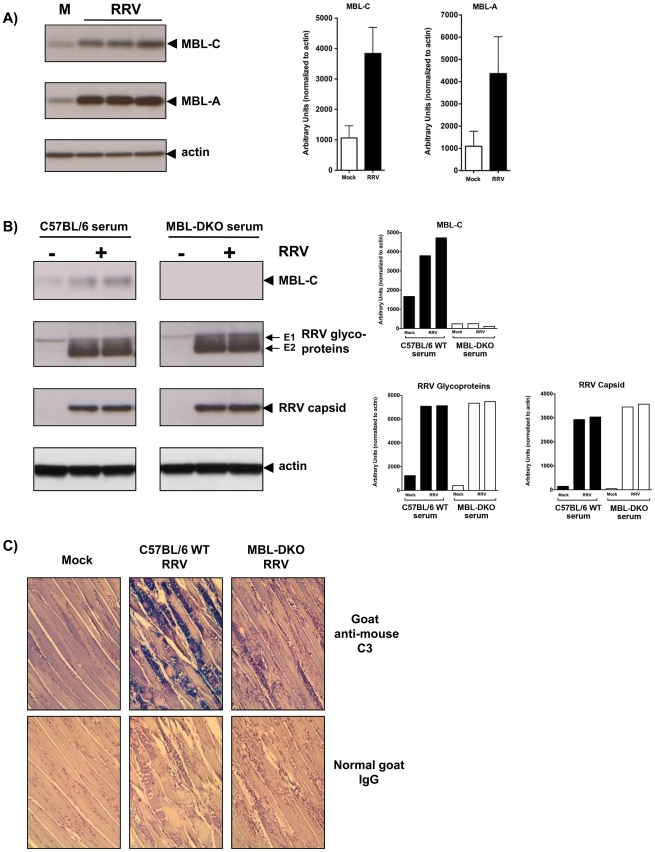
RRV infection induces MBL deposition onto cells, resulting in MBL-dependent C3 deposition onto infected tissues. (A) To determine if MBL levels are elevated within the quadriceps muscles of RRV-infected wild-type animals, homogenized quadriceps muscles from either mock- or RRV-infected WT mice at 7 dpi were analyzed by immunoblot analysis using anti-mouse MBL-A, anti-mouse MBL-C, or anti-mouse actin antibodies. Each lane represents an individual mouse and is representative of at least three independent experiments. Densitometry measurements of bands in immunoblot from three different experiments are graphically depicted as arbitrary units normalized to actin (mock n = 4; RRV n = 9). (B) To determine if MBL deposition is enhanced onto RRV-infected cell, differentiated C2C12 murine skeletal muscle cells were infected with RRV, and incubated with either serum from a WT or MBL-DKO mouse for 30 minutes prior to harvesting. Cells were washed, harvested, and lysates were analyzed by immunoblot analysis using anti-mouse MBL-C, anti-RRV, or anti-mouse actin antibodies. Densitometry measurements of bands in immunoblot are graphically depicted as arbitrary units normalized to actin (C) C3 deposition was assessed by IHC using an anti-mouse C3 antibody on quadriceps muscle sections from either mock- or RRV-infected WT or MBL-DKO mice at 7 dpi. C3 positive areas are stained in blue. A representative section from each strain is shown and is representative of two independent experiments. No signal was observed in sections incubated with a control goat IgG antibody.

To determine if MBL is deposited onto tissues following RRV infection, we evaluated tissue homogenates of quadriceps muscle from RRV-infected WT mice at 7 dpi for levels of MBL by immunoblot analysis. We observed an increase in the amount of MBL in the quadriceps muscle of infected mice compared to that of mock-infected mice (Figure 2A), indicating that RRV infection results in elevated amounts of MBL within target tissues. Given the enhanced MBL within RRV infected muscle tissue, we next evaluated whether MBL would directly bind to RRV or RRV infected cells. Studies with two other alphaviruses, CHIKV and Sindbis virus, found no evidence for interactions with MBL, while mosquito derived West Nile virus was efficiently bound and neutralized by MBL [18]. Consistent with these findings, we were unable to detect direct binding between MBL and either mammalian cell or mosquito cell derived RRV virions by ELISA (data not shown) and we also found no evidence for MBL-mediated neutralization of RRV (Figure S2). Given the lack of detectable interactions between MBL and the RRV virion, we assessed whether MBL bound to RRV-infected cells. Differentiated C2C12 murine skeletal muscle cells were infected with RRV for 24 hours and then incubated with medium containing either C57BL/6 wild-type serum or MBL-DKO serum for 30 minutes. Cell lysates were harvested and analyzed for presence of MBL-C by immunoblot analysis. As shown in Figure 2B, the amount of MBL-C deposition was enhanced in cells infected with RRV compared to mock infected cells, indicating that RRV infection results in increased MBL binding to cells. No deposition of MBL was detected onto cells incubated with MBL-DKO serum, indicating the specificity of detection. Therefore, though MBL does not appear to interact with the RRV virion, RRV infection does lead to MBL deposition on infected cells.

### Complement deposition and activation is reduced in RRV-infected MBL-DKO compared to WT mice

MBL, but not the alternatively or classical complement activation pathways, was required for RRV-induced disease and tissue pathology (Figure 1), and the phenotype in MBL-DKO mice is strikingly similar to C3^−/−^ mice, suggesting that MBL plays a major role in driving complement activation during RRV infection. Therefore, we directly assessed whether MBL was required for RRV-dependent complement deposition. Western blot analysis of skeletal muscle from wild type or MBL-DKO mice indicated that C3 levels, including the α and β chains of C3 were present at reduced levels in the skeletal muscle of RRV infected MBL-DKO mice compared to wild type mice (Figure S3A–B). However, since inflammatory macrophages produce C3 [22], western blot analysis was not able to clearly differentiate between complement deposition within the tissue and de novo production of complement by the infiltrating inflammatory cells in both wild type and MBL-DKO animals. Therefore, we directly assessed the impact of MBL deficiency on complement deposition within the RRV infected muscle by performing immunohistochemistry on quadriceps muscle from WT and MBL-DKO mice using an anti-mouse C3 antibody. Abundant C3 staining localized to damaged skeletal muscle at 7 dpi in WT mice while C3 staining was substantially reduced in muscle from RRV-infected MBL-DKO mice (Figure 2C). Importantly, we observed comparable C3 staining between RRV-infected C1q^−/−^, fB^−/−^, and WT mice (Figure S3C), suggesting that neither C1q nor fB are required for C3 deposition on muscle tissue following RRV infection. Therefore, these results suggest that MBL is the major mediator of complement activation and deposition within RRV infected muscle tissue.

### Viral replication is unaffected in the muscle tissue of MBL-DKO mice

**Figure 3:**
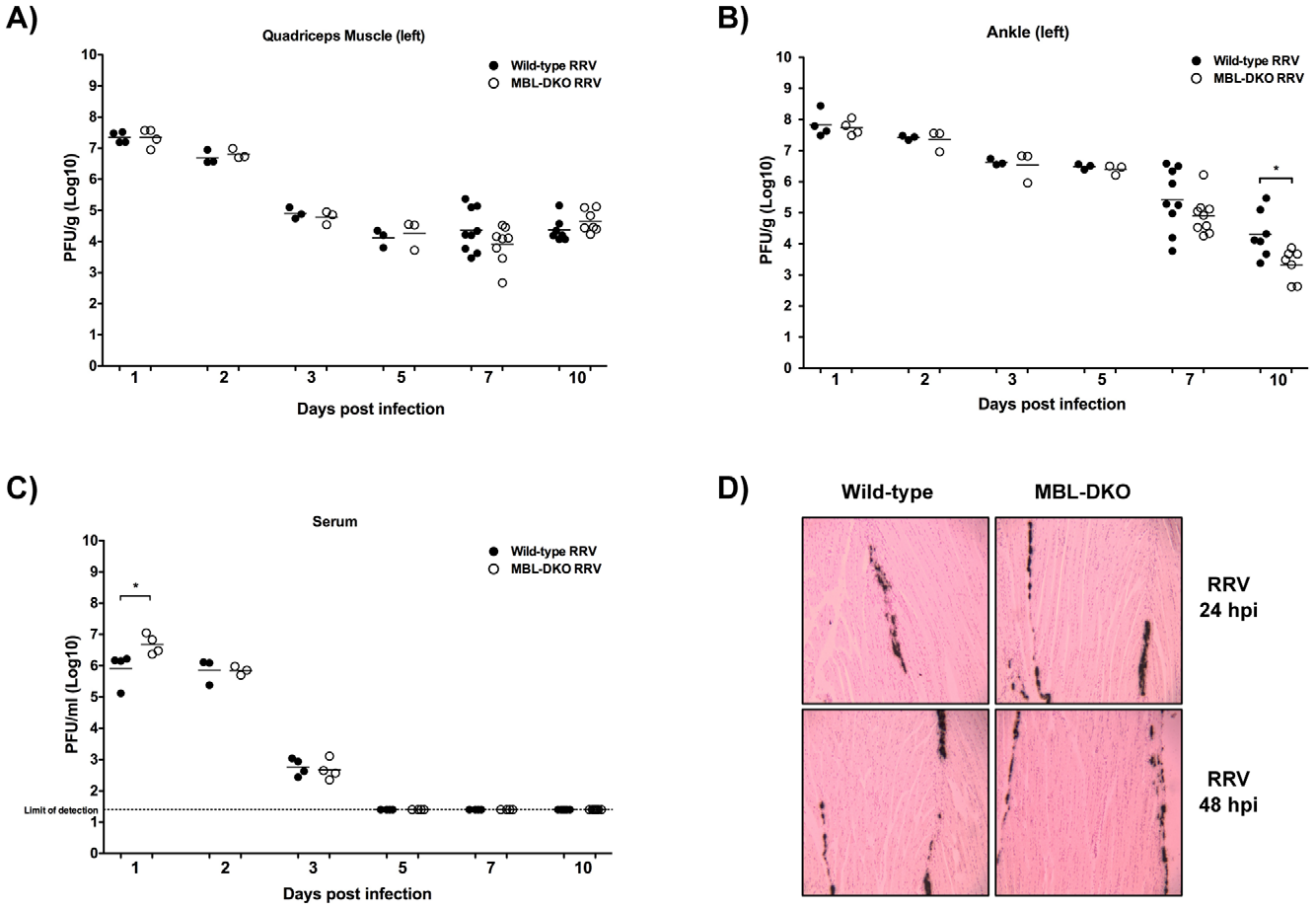
MBL deficiency does not affect viral replication or tropism within infected tissues. (A–C) Quadriceps muscle (A), ankle joints (B), and serum (C) from RRV-infected WT (solid circles, n = 3–9/time point) or MBL-DKO (open circles, n = 3–8/time point) mice were assayed to determine viral titer at various times post infection. Viral titer was determined by plaque assay on BHK-21 cells. Each data point represents the viral titer from a single animal; data is combined from two independent experiments. *p<0.05 as determined by t-test. (D) Tropism within the quadriceps muscle tissue was determined by *in situ* hybridization using RRV-specific probe. We did not detect any signal using an EBER-specific probe (data not shown). A representative section from each strain is shown (n = 3 for both WT and MBL-DKO mice).

Prior studies with C3^−/−^ and CR3^−/−^ mice demonstrated that complement activation and CR3-dependent signaling is essential for RRV-induced disease and tissue destruction, but complement deficiency had no effect on viral burden or tropism. To determine whether this was also the case in MBL-DKO mice, we evaluated WT and MBL-DKO mice for viral load within the quadriceps muscles, ankle joints, and serum. As shown in Figure 3A, MBL-DKO mice exhibited no significant difference in the amount of infectious virus in the quadriceps muscle through days 7 and 10 dpi, which represent the times when RRV-induced muscle destruction peaks. Furthermore, analysis of the viral distribution within wild type and MBL-DKO animals by in situ hybridization found no differences in the localization of RRV specific signal between the two mouse strains. (Figure 3D). Therefore, the differences in RRV-induced tissue destruction (Figure 1C–D) or C3 deposition (Figure 2C) within the RRV infected muscle of MBL-DKO mice cannot be explained by differences in viral replication.

In addition to evaluating viral titers within the skeletal muscle, we also assessed viral loads within the serum and ankle joints. Viral titers within the ankle joints were similar between MBL-DKO and WT mice through 7 dpi (Figure 3B), though we did observe a small, but statistically significant decrease in viral titer within the ankle joints of MBL-DKO mice compared to wild type mice at day 10 post infection. MBL-DKO mice had higher amounts of virus in the serum at 1 dpi compared to WT mice (Figure 3C), indicating that MBL may play some role in initial control of viremia. However, virus was cleared from the serum of infected animals at similar rates in both WT and MBL-DKO mice (Figure 3C), suggesting that MBL does not play a major role in serum clearance of RRV or direct neutralization of virus in the serum. The impact of this initial increase in serum viremia in MBL-DKO mice on downstream disease through antibody production is unclear, although it is important to note that both RAG-1^−/−^ and μMT mice develop disease similar to WT mice [8], indicating that the antibody response is not required for development of disease.

### MBL deficiency does not affect inflammatory cell recruitment, but alters expression of inflammatory mediators within the RRV-infected muscle

**Figure 4:**
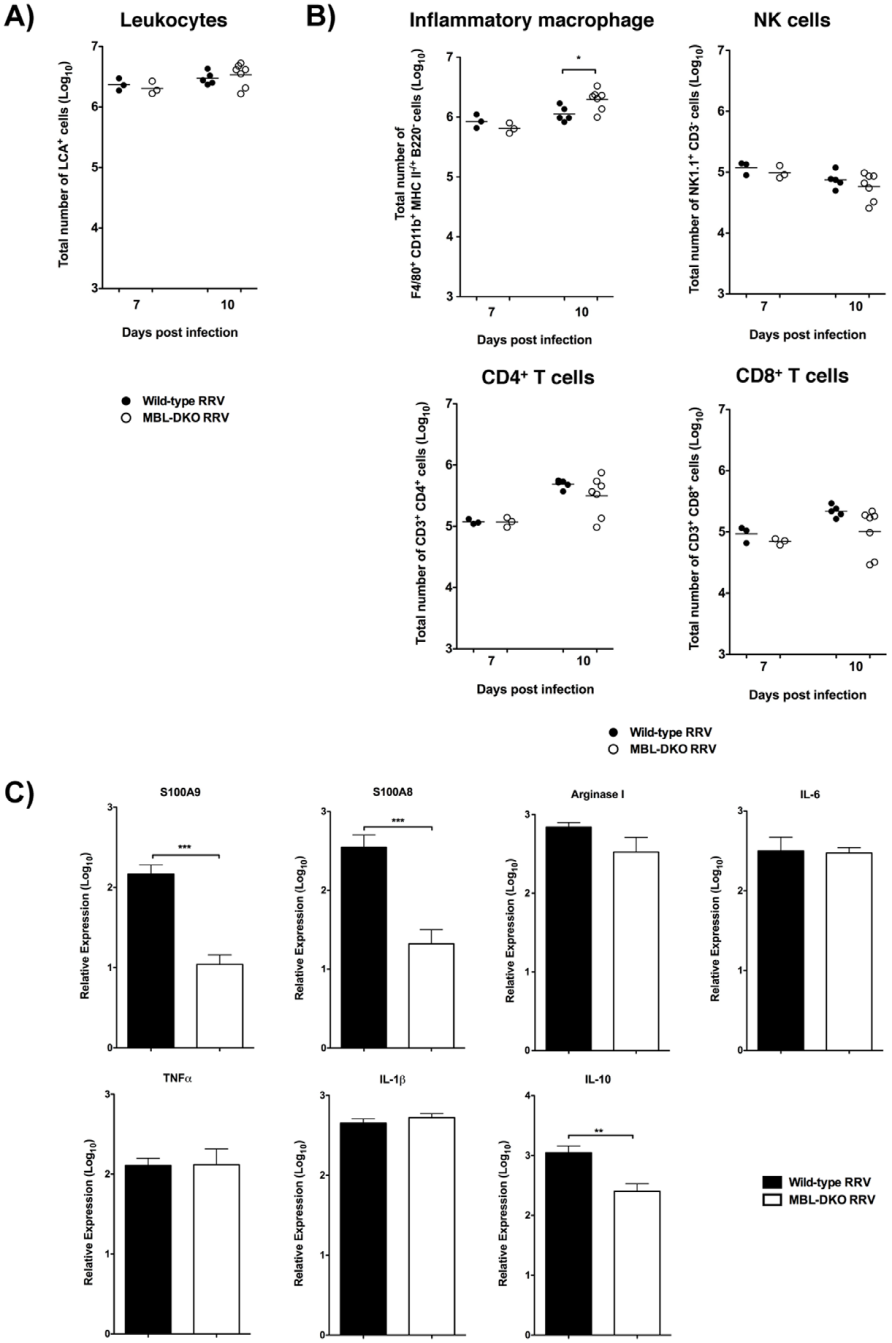
MBL deficiency does not affect inflammatory cell recruitment, but alters expression of inflammatory mediators within the RRV infected muscle. (A–B) Leukocytes were isolated from the quadriceps muscle of RRV-infected WT (solid circles, n = 3–5/time point) or MBL-DKO (open circles, n = 3–7/time point) mice at 7 or 10 dpi. Cells were characterized and quantified by flow cytometry using the markers described in the materials and methods. Total numbers of leukocytes (A) and specific cells types (B) are shown. A single experiment for each time point is shown. Compiled data for three independent experiments is shown in Fig. S4 (7 dpi). *p<0.05 by Mann-Whitney analysis. (C) Relative mRNA expression of C3-dependent inflammatory mediators and cytokines S100A9, S100A8, Arginase I, and IL-6 (top panel) and C3-independent cytokines TNFα, IL-1β, and IL-10 (bottom panel) from quadriceps muscle from RRV-infected WT (solid bar, n = 3) or MBL-DKO (open bar, n = 3) mice by quantitative real-time PCR. Raw data values were normalized to 18S rRNA levels, log-transformed, and are graphically depicted as fold expression over mock-infected mice. Data from a single experiment is shown, but is representative of two independent experiments. ***p<0.001; **p<0.005 by t-test.

Prior studies demonstrated that complement activation drives inflammatory tissue destruction, but does not regulate inflammatory cell recruitment during RRV infection [8]. However, as MBL may regulate the host inflammatory response independently of its effects on complement activation, we quantified and analyzed the inflammatory cell populations within the muscle of WT and MBL-DKO animals at 7 and 10 dpi, which are the times of peak inflammation in WT mice [20]. Consistent with prior findings in C3^−/−^ mice, RRV infected MBL-DKO mice exhibited no statistically significant differences in either total number of leukocytes (Figure 4A and Figure S4A) or the composition of the inflammatory infiltrates at either 7 or 10 dpi (Figure 4B and Figure S4B). Representative flow cytometry plots of the various cell types are shown in Figure S5. The total numbers of CD4^+^ T cells, CD8^+^ T cells, and NK cells at both 7 and 10 dpi were not significantly different between RRV-infected WT and MBL-DKO mice (Figure 4B and Figure S4B). Given the role of inflammatory macrophage in development of RRV-induced disease [6], we compared total numbers of cells with staining characteristics of inflammatory macrophages (F4/80^+^ CD11b^+^ Gr-1^lo^ B220^−^) at both 7 and 10 dpi, and observed no difference at 7 dpi, and interestingly, a significant increase in numbers of these cells in RRV-infected MBL-DKO mice at 10 dpi. While we cannot rule out the possibility that minor populations of inflammatory cells are differentially regulated by MBL, these data suggest that MBL does not affect the major populations of inflammatory infiltrates recruited to the skeletal muscle following RRV infection.

Although inflammatory cell recruitment was largely unaffected by either MBL or C3 deficiency [8] we have previously shown CR3 is also required for RRV-induced disease and that a subset of inflammatory genes expressed in the inflamed muscle of RRV-infected mice are dependent upon both C3 and CR3, including the calgranulins S100A8 and S100A9, IL-6 and the enzyme arginase I (ArgI) [9]. Therefore, to determine if MBL affected expression of these genes in the same manner as C3 and CR3, expression levels were assessed in the quadriceps muscle from both WT and MBL-DKO mice at 7 dpi. As shown in Figure 4C, RRV-infected WT mice exhibited significantly higher expression of S100A8 and S100A9 compared to RRV-infected MBL-DKO mice, indicating that expression of these genes is also regulated by MBL during RRV infection. Interestingly, the S100A8/S100A9 complex has been associated with inflammatory arthritis (reviewed in [23]) however, the role of these proteins in RRV disease requires further investigation. Expression of TNFα and IL-1β, which were shown to be C3-independent [9], were also unaffected in MBL-DKO mice (Figure 4C). Expression of IL-6 and Arg I, which we have previously shown to be C3 and CR3-dependent, were unaffected in MBL-DKO mice (IL-6) or slightly reduced (Arg I) (Figure 4C). Expression of these genes may reflect residual complement activation in the absence of MBL (Figure S3), though this requires further study. Interestingly, expression of IL-10, which is largely C3-independent following RRV infection [9], was dependent on MBL and suggests that MBL may be interacting with pathways other than the complement system to mediate IL-10 expression (Figure 4C).

### Levels of MBL are elevated in RRV patients

**Figure 5:**
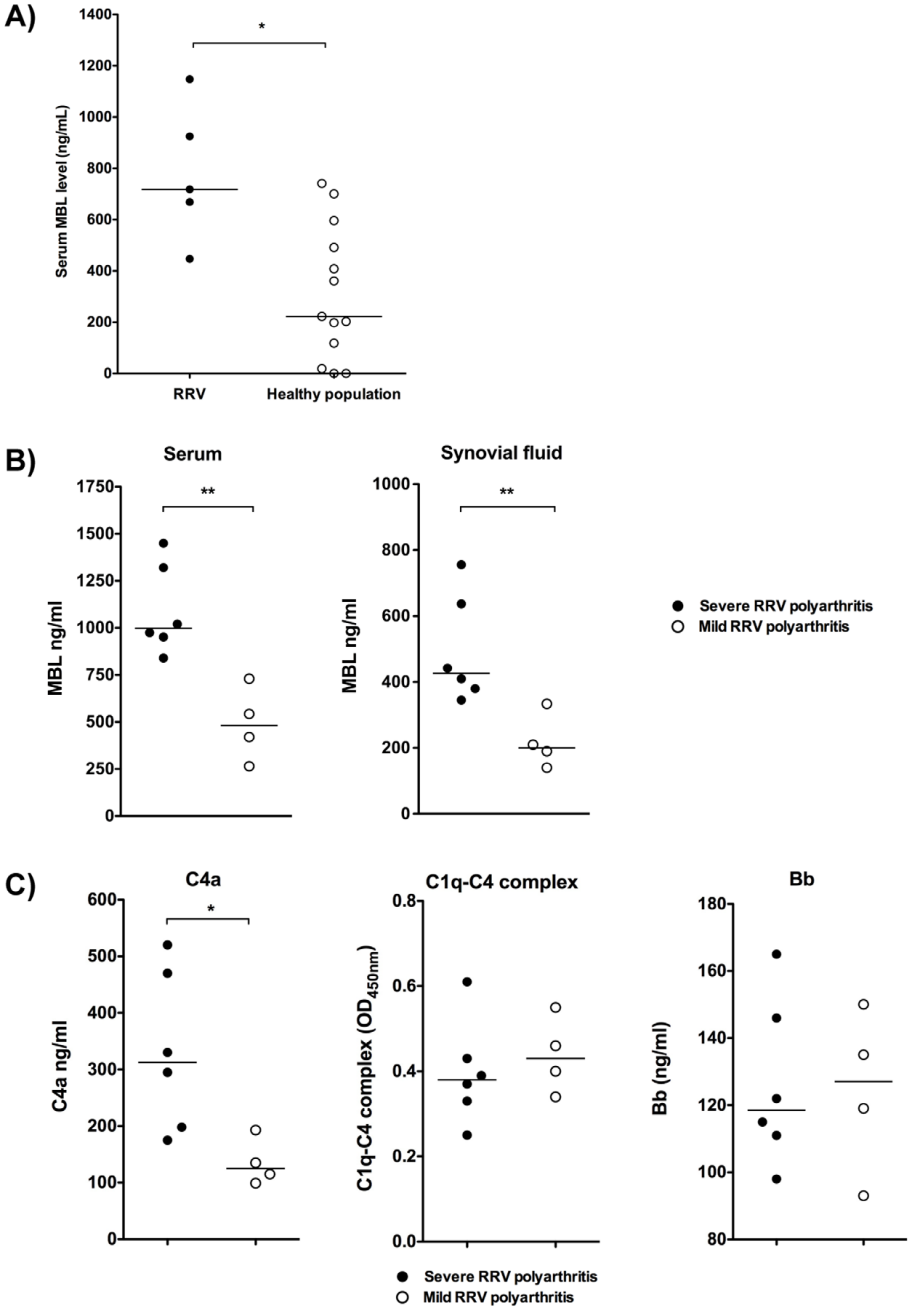
Levels of MBL are elevated in RRV patients. (A) Serum from RRV-infected patients (solid circles, n = 5) or healthy sero-negative controls (open circles, n = 13) were analyzed by ELISA for MBL levels. Each data point represents a single individual, and bar represents the median. *p<0.05 by Mann-Whitney analysis. (B) MBL levels within serum and synovial fluid from patients clinically characterized with either severe RRV-induced disease (solid circles, n = 6) or mild RRV-induced disease (open circles, n = 4) were analyzed by ELISA. Each data point represents a single individual, and the bar represents the median. **p<0.01 by Mann-Whitney analysis. (C) Levels of C4a (left), C1q-C4 complex (middle) and Bb (right) within the synovial fluid from patients described in (B) were analyzed by ELISA. *p<0.05 by Mann-Whitney analysis.

Prior studies have shown that the C3 cleavage product C3a is elevated in synovial fluid of RRV polyarthritis patients [8]. To determine if circulating levels of MBL are elevated in RRV-infected patients, we compared serum MBL levels from patients during convalescence to serum MBL levels in RRV-seronegative controls. As shown in Figure 5A, RRV patients had significantly higher levels of circulating MBL compared to healthy controls. Since MBL levels are highly variable in human populations, we also assessed serum and synovial fluid MBL levels in a small cohort of patients clinically characterized as having severe or mild RRV-induced polyarthritis. MBL levels correlated with severity of RRV disease (Figure 5B), with higher levels of MBL observed in patients classified as having severe disease. Severity of disease also correlated with increased levels of C4a in the synovial fluid (Figure 5C), which could result from complement activation through either the lectin or classical pathway. However, analysis of the level of C1q-C4 complexes formed within the synovial fluid, an activation marker of the classical pathway [24], showed no difference between patients with severe or mild disease (Figure 5C), suggesting that the higher C4a levels in severe RRV disease was primarily due to activation through the lectin pathway. In addition, we did not observe a difference in levels of Bb, an activation marker of the alternative pathway (Figure 5C), further supporting the hypothesis that the MBL pathway primarily mediates complement activation following RRV infection. Importantly, when we assessed MBL levels in a cohort of patients suffering from non-inflammatory osteoarthritis, we found no evidence for elevated MBL levels (mean MBL levels of 57.8±24.8 ng/ml [n = 5 patients with severe osteoarthritis]) compared to MBL levels of 485±163.7 ng/ml in patients with severe RRV induced disease and 218.5±82.4 ng/ml within the synovial fluid of patients with mild RRV-induced disease, suggesting that elevated levels of MBL are not simply the result of arthritis symptoms within the joints. Although additional studies with a larger cohort of patients are required to determine whether MBL levels associate with the severity of RRV-induced arthritis, and whether this effect reflects a causal role for MBL in human disease, these results, combined with the knockout mouse studies strongly suggest that the MBL pathway of complement activation plays a major role in the pathogenesis of RRV-induced inflammatory disease.

## Discussion

Alphaviruses such as CHIKV and RRV represent significant emerging disease threats that cause large-scale outbreaks of severe chronic and persistent arthralgia/myalgia in human populations. Though alphavirus-induced arthralgia and myalgia is often debilitating, the mechanisms by which these viruses cause arthritis/arthralgia are not fully understood. Previous studies have shown that inflammatory macrophages play a major role in the pathogenesis of both RRV and CHIKV [6], [25], that the host complement cascade is essential for the induction of muscle destruction by these inflammatory cells during RRV infection, and that this process is dependent on complement receptor 3 (CR3) [8], [9]. The data presented here demonstrate that RRV infection results in the deposition of MBL within RRV infected tissues, and that MBL, but not the alternative or classical complement activation pathways, was essential for RRV induced complement activation and subsequent inflammatory tissue destruction and disease. Consistent with this, humans suffering from severe RRV disease exhibited increased levels of MBL, but not markers of the classical or alternative complement activation pathways in their synovial fluid. Therefore, these studies demonstrate that MBL plays a key role in promoting the pathogenesis of alphavirus-induced inflammatory disease, and suggest that MBL may represent a target for therapeutic intervention in the treatment of alphavirus-induced arthritis/myositis.

MBL has generally been associated with a protective role during viral infection, either through its ability to neutralize viruses directly or via the downstream activation of complement. In the context of arbovirus infection, MBL contributes to direct neutralization of mosquito-derived West Nile virus and both mammalian and mosquito-derived dengue virus through interactions between MBL and viral N-linked glycans [18], [26], and MBL contributes to protection from West Nile virus-induced disease in vivo [19]. Though the host complement cascade has been shown to play a protective role during neurotropic alphavirus infection [21], [27], [28] these processes are dependent upon either the classical or alternative complement activation pathways [21]. Furthermore, though Fuchs, et al., found that MBL bound and neutralized WNV virions, they found no evidence for MBL interactions with two alphaviruses, CHIKV and Sindbis virus [18], which is supported by our inability to demonstrate direct binding or neutralization of RRV virions by MBL. Therefore, our findings demonstrate a novel role for MBL in the pathogenesis of alphavirus-induced arthritis/myositis and indicate that this pathway, which plays a protective role against many viral infections, is actually a major driver of RRV-induced tissue pathology and disease.

In addition to its prominent role in the pathogenesis of RRV-induced disease, the complement cascade is linked to a number of host autoimmune inflammatory disorders, including rheumatoid arthritis (reviewed in [29]). However, the role of MBL in these processes is less clear. Though MBL has been shown to contribute to ischemic injury in mouse models of cardiac or intestinal reperfusion injury [12]–[15], MBL has not been directly linked to inflammatory arthritis. There are conflicting reports associating MBL polymorphisms with rheumatoid arthritis in humans [30]–[34], however in mouse models of collagen-induced arthritis, which serves as a model of RA, MBL is dispensable for complement activation and arthritis induction [35], [36]. Therefore, MBL appears to be playing a unique role in the pathogenesis of RRV-induced arthritis/myositis that is not shared with other arthritic syndromes, though further comparisons between these different disease states are needed to clarify this issue.

The studies presented here demonstrate that MBL-dependent complement activation promotes RRV-induced disease and raises several questions relating to the mechanism of RRV activation of complement, the role of MBL polymorphisms in determining disease severity, and the therapeutic potential of MBL inhibition to treat RRV-infected patients. The CRD of MBL recognizes terminal carbohydrates, such as mannose and glucose, which can be found on glycosylated proteins in bacteria and viruses. The RRV glycoproteins contain three N-linked glycosylation sites that are glycosylated with a combination of high mannose and complex glycans [37] and may serve as ligands for MBL, leading to complement activation. While we did not observe direct binding of MBL to virions, we did observe an increase in the amount of MBL deposited onto infected tissues and on virally infected cells (Figure 2), indicating that some aspect of RRV infection induces MBL deposition and complement activation. Alphaviruses bud from the plasma membrane of infected cells and the viral glycoproteins are prominently exposed on the surface of the cell. Therefore, it is possible that MBL is recognizing and binding to viral glycoproteins on infected tissues during viral egress, resulting in complement activation directly onto the tissue rather than binding to free virus. Alternatively, viral infection may lead to the modification of host cell N-linked glycans or other cellular components, thereby promoting MBL deposition and complement activation; however, both of these possibilities require further investigation.

Given the central role of MBL in development of severe RRV-induced disease, specific inhibition of the MBL activation pathway of the complement system in RRV-patients may be a strategy to alleviate disease. Current therapy involves administration of non-steroidal anti-inflammatory drugs, and given the role that complement plays in mediating severe RRV-induced disease, treatment with complement inhibitors may provide an attractive alternative to nonspecific anti-inflammatory drugs. However, prolonged inhibition of the complement system can leave patients susceptible to other infectious diseases, especially as treatment of disease symptoms may require several months for some individuals [38]. Our results suggest that a more focused approach targeting MBL may prove effective in limiting RRV-induced arthralgia/myalgia, while limiting the general immune suppression associated with complement inhibition. Inhibitors targeting the MBL pathway of complement through inhibition of MASP-2 are in development [39], and may be useful in treatment of RRV-induced disease in humans.

In addition to raising the possibility of targeting MBL in the treatment of RRV or other alphavirus-induced arthraligias/myalgias, these studies raise the issue of whether polymorphisms in MBL affect susceptibility to RRV-induced disease. Common genetic polymorphisms within the promoter region and exon 1 of the human *Mbl2* gene lead to variations in serum MBL levels or functional deficiency of MBL (reviewed in [40]). Human patients with severe RRV disease have higher levels of MBL within the synovial fluid and serum; however, it is unclear if levels of MBL are elevated in response to severe RRV infection or if naturally higher levels of MBL contribute to the development of severe disease. Preliminary analysis of a small cohort of RRV patients does not associate *Mbl2* polymorphisms with severity of RRV disease (S. Mahalingam, B. Piraino, B. Cameron, L. Herrero, and A. Lloyd, unpublished data), however a larger cohort of RRV-infected individuals must be analyzed before we can conclude whether MBL polymorphisms associate with RRV-induced disease severity or if up-regulation of MBL in response to viral infection contributes to disease pathogenesis in humans.

In summary, the data presented in this study demonstrate the role for MBL in promoting severe disease following RRV infection through complement activation and subsequent destruction of RRV infected tissue. Numerous studies have shown a protective role for MBL and the complement system in response to a diverse set of viruses. Our results demonstrate a novel role of MBL following viral infection in which MBL contributes to development of severe disease, and these findings suggest that MBL may be a therapeutic target for treatment in humans suffering from RRV-induced polyarthritis or other alphavirus-induced arthritides.

## Materials and Methods

### Ethics statement

Some of the studies described in this manuscript did involve human samples. For human serum samples, all serum samples had been submitted for diagnostic testing with written and oral informed patient consent at CIDMLS, Westmead Hospital and The Royal Melbourne Hospital (Melbourne, Australia). Samples were de-identified by the testing laboratory before being used in the research project. Synovial samples were collected from adult patients (age range, 30–45 years) residing in the Murray-Goulburn Valley (Victoria, Australia) who had acute cases of RRV-induced polyarthritis in accordance with human subjects protocols approved by the Royal Melbourne Hospital Human Ethics Committee. All individuals received and completed written informed consent forms prior to collection of materials. Mouse studies were performed in strict accordance with the recommendations in the Guide for the Care and Use of Laboratory Animals of the National Institutes of Health. All mouse studies were performed at the University of North Carolina (Animal Welfare Assurance # A3410-01) using protocols approved by the UNC Institutional Animal Care and Use Committee (IACUC). All studies were performed in a manner designed to minimize pain and suffering in infected animals, and any animals that exhibited severe disease signs was euthanized immediately in accordance with IACUC approved endpoints.

### Viruses and cells

The viral stocks used in this study were generated from the infectious clone of the T48 strain of RRV (pRR64), kindly provided by Richard Kuhn (Purdue University) as described in [20]. Briefly, viral RNA was generated through *in vitro* transcription of SacI-linearized pRR64 using the mMessage mMachine SP6 kit (Ambion) and electroportated into BHK-21 cells (ATCC). Viral titer was determined by plaque assay on BHK-21 cells. BHK-21 cells were grown in α-MEM (Gibco) supplemented with 10% donor calf serum (DCS), 10% tryptose phosphate, L-glutamine, penicillin, and streptomycin. C2C12 cells were grown in DMEM (Gibco) supplemented with 20% fetal bovine serum (FBS), L-glutamine, penicillin, and streptomycin prior to differentiating. To differentiate cells into myotubes, confluent C2C12 cells were maintained in DMEM supplemented with 2% horse serum, L-glutamine, penicillin, and streptomycin.

### Mice

All mice used in this study were maintained and bred in house at the University of North Carolina (UNC) in accordance with UNC Institutional Animal Care and Use Committee guidelines. C57BL/6 and MBL-DKO mice were purchased from The Jackson Laboratories (Bar Harbor, ME); C1q^−/−^ mice were a generous gift from Dr. Marina Botto (Imperial College London, UK); fB^−/−^ mice were generously provided by Dr. Charles Jennette (UNC). While RRV is classified as a biosafety level-2 agent, due to the exotic nature of the virus, all animal studies were performed in a biosafety level-3 facility. Twenty-four day old mice were inoculated with 10^3^ PFU of RRV in diluent (phosphate buffered saline supplemented with 1% DCS) into the left rear footpad. Mice were weighed daily and assigned a clinical score based on hind limb weakness and altered gait on the following scale: 0 = no disease; 1 = mild loss of hind limb grip; 2 = moderate loss of hind limb grip; 3 = severe loss of hind limb grip; 4 = no hind limb grip and mild inability to right; 5 = no hind limb grip and complete inability to right; 6 = moribund.

### Viral burden analysis

Mice were infected with RRV as described above, and at indicated times post infection mice were sacrificed, perfused with 1× PBS, and indicated tissues were dissected out, weighed, and homogenized with glass beads in diluent. Viral titer within infected tissues were determined by plaque assay on BHK-21 cells from tissue homogenates.

### *In situ* hybridization

Mice were infected with RRV as described above, and at indicated times post infection mice were sacrificed, perfused with 4% paraformaldehyde (PFA), pH 7.3. Tissues were paraffin embedded and 5 µm sections were generated and in situ hybridization was performed as previously described [20] using an RRV-specific or EBER-specific ^35^S-labeled RNA probe.

### Histological analysis

At desired times post infection, mice were sacrificed and perfused with 4% paraformaldehyde (PFA), pH 7.3. Tissues were paraffin embedded and 5 µm sections were generated and stained with hematoxylin and eosin (H&E) to examine tissue pathology and inflammation. Sections were visualized by bright field light microscopy (Olympus BX61).

### Evans blue dye uptake analysis

At 10 days post infection mice were injected with 1% Evans blue dye in PBS into the peritoneal cavity (50 µl/10 g mouse weight). At 6 hours post injection, mice were sacrificed and perfused with 4% PFA. Quadriceps muscle tissues were embedded in optimal cutting temperature compound (OCT) and frozen in an isopentane histobath, 5 µm sections were generated, mounted with ProLong Gold with DAPI (Invitrogen) and sections were analyzed by fluorescence microscopy (Olympus BX61).

### Immunohistochemistry

At 7 dpi, mice were sacrificed and perfused with 4% PFA. Quadriceps muscles were removed, paraffin embedded, and 5 µm sections were generated. Sections were deparaffinized in xylene, rehydrated through an ethanol gradient, and probed with a goat anti-mouse C3 polyclonal antibody (1∶500 Cappel) using the Vectastain ABC-AP kit (Vector Labs, CA) and Vector Blue Alkaline phosphatase substrate kit (Vector Labs, CA) according to the manufacturers' instructions. Sections were counterstained with Gill's hematoxylin.

### Immunoblot analysis

At indicated times post infection, mock-infected and RRV-infected mice were sacrificed, and perfused with 1× PBS. Quadriceps muscles were removed and homogenized in radioimmunoprecipitation lysis buffer (RIPA; 50 µM Tris pH 8.0, 150 mM NaCl; 1% NP-40, 0.5% deoxycholate, 0.1% SDS and 1× complete protease inhibitor cocktail (Roche)) by glass beads. Protein concentration was determined by Bradford protein assay and 25–30 µg of protein was run onto a 10% SDS-PAGE gel. Protein was transferred onto a PVDF membrane, and membranes were blocked in 5% milk, 0.1% Tween-20 in PBS. Membranes were probed with goat anti-mouse MBL-A (1∶1000 R&D Systems), goat anti-mouse MBL-C (1∶1000 R&D Systems), goat anti-mouse C3 polyclonal antibody (1∶1000 Cappel), mouse anti-RRV (1∶1000 ATCC), or goat anti-mouse actin polyclonal antibody (1∶500 SCBT), washed with PBS containing 0.1% Tween-20 and incubated with rabbit anti-goat antibody or sheep anti-mouse antibody conjugated to horseradish peroxidase (1∶ 10, 000 Sigma). Membranes were washed again and protein visualized by ECL (Amersham) according to manufacturer's instructions. Densitometry was performed using ImageJ software (NIH).

### MBL deposition onto C2C12 cells

Differentiated C2C12 cells were either mock-infected or infected at an approximate MOI of 20 with RRV. At 24 hpi, culture medium was removed and cells were incubated in differentiation medium containing either 10% serum from WT or MBL-DKO mice for an additional 30 minutes. Cells were washed and harvested in RIPA lysis buffer, and cell lysates were analyzed by immunoblot analysis.

### Analysis of infiltrating inflammatory cells by flow cytometry

To determine the composition of the inflammatory cell infiltrates within the quadriceps muscle, at indicated times post infection mice were sacrificed and perfused with 1× PBS. Both quadriceps muscles were removed, minced, and digested with RPMI containing 10% fetal bovine serum (FBS), 15 mM HEPES, 2.5 mg/ml collagenase A (Roche), 1.7 mg DNase I (Roche) for 2 hours at 37°C with shaking. Cells were strained through a 40 µm strainer and washed twice with wash buffer (HBSS containing 1% sodium azide and 1% FBS) and total viable cells were determined by trypan blue exclusion. To stain cells for flow cytometry, cells were incubated with anti mouse FcgRII/III (2.4G2; BD Pharmingen) and stained with combinations of the following antibodies: fluorescein isothiocyanate (FITC)-conjugated anti-mouse CD3, phycoerythrin (PE)-conjugated anti-NK1.1, PE-Cy5 anti-CD45 (leukocyte common antigen), PE-Cy7 anti-F4/80, Allophycocyanin (APC)-conjugated anti-CD49b, eF450-conjugated anti-CD11b, APC anti-major histocompatibility complex class II antigens (MHC II), and eF780-conjugated anti-CD45 (B220) (eBiosciences, San Diego, CA), FITC anti-Ly-6G, and PE anti-SigLecF (BD-Pharmingen, San Diego, CA), and PE-Texas Red-conjugated anti-CD45 (B220), and PE-Texas Red anti-CD11c (Molecular Bioprobes, Invitrogen). Cells were fixed with 2% PFA (pH 7.3) and analyzed on a CyAn flow cytometer (Becton Dickinson), and data was analyzed using Summit software.

### Gene expression

At 7 dpi following RRV infection, mice were sacrificed and perfused with 1× PBS. Quadriceps muscles were removed and homogenized in Trizol (Invitrogen) using glass beads. RNA was extracted using Invitrogen PureLink RNA purification kit, and mRNA expression of indicated genes was measured by quantitative real-time PCR. Raw data values were normalized to 18S rRNA levels.

### Patient samples

Convalescent serum samples from five patients presenting with acute, serologically confirmed (seroconversion by neutralization, IgM and IgG) RRV-infection and thirteen samples from healthy individuals were provided by CIDMLS, Westmead Hospital (Sydney, Australia). All serum samples had been submitted for diagnostic testing with informed patient consent at CIDMLS, Westmead Hospital and The Royal Melbourne Hospital (Melbourne, Australia). Samples were de-identified by the testing laboratory before being used in the research project. Needle biopsy was performed to collect synovial fluid samples from adult patients (age range, 30–45 years) residing in the Murray-Goulburn Valley (Victoria, Australia) who had acute cases of RRV-induced polyarthritis. Samples were collected and prepared aseptically in the laboratories of Echuca Hospital (Murray-Goulburn Valley; Victoria, Australia) and The Royal Melbourne Hospital and was performed in accordance with The Royal Melbourne Hospital Human Ethics Committee. Severe RRV-induced disease was defined as a patient presenting with intense swelling, severe joint pain and myalgia affecting both the knee joints and joints of the fingers. Mild RRV-induced disease was defined as a patient presenting with minor swelling, localized in the knees, and no additional symptoms. For osteoarthritis samples, synovial fluid aspirates were obtained from 5 patients with osteoarthritis from the John James Hospital (Canberra, Australia). Sample collection was performed in accordance with the AustralianCapital Territory Health Community Care Human Research Ethics committee. Samples were obtained at the time that knee joint arthroplasty was performed, and joints were aspirated before arthrotomy. The diagnosis given to patients was primary osteoarthritis with no evidence of an inflammatory arthropathy. These samples were de-identified prior to analysis.

Levels of MBL in serum and synovial fluid were determined using a commercially available ELISA kit according to the manufacturer's instructions (R&D Systems). Levels of C4a in the synovial fluid was determined using BD OptEIA (BD). Bb levels were determined using Microvue Bb Plus (Quidel). The levels of the C1q-C4 complex were determined as described in [24].

### Statistical analysis

Clinical scores and percent of starting weight at 10 dpi between C1q^−/−^, fB^−/−^, MBL-DKO, and wild-type mice were analyzed for statistically significant differences by Mann-Whitney analysis with multiple comparisons corrections (clinical scores; p<0.01 is considered significant), and by one-way ANOVA with Bonferroni's correction (percent of starting weight; p<0.05 is considered significant). Viral burden, total number of infiltrating cells, and gene expression data at each time point between wild-type and MBL-DKO mice was analyzed for statistically significant differences by Mann-Whitney analysis or t-test (p<0.05 is considered significant). Levels of MBL, C4a, C1q-C4 complexes, and Bb in serum and synovial fluid from human patients were analyzed by Mann-Whitney analysis for statistical significance (p<0.05 is considered significant). Statistical analyses were performed using GraphPad Prism 5.

## Supporting Information

Figure S1 **C57BL/6 wild-type and MBL-DKO mock-infected control mice exhibit weight gain throughout course of experiments.** Twenty-four day old WT C57BL/6 (solid circle, n = 5–13/time point) or MBL-DKO (open circle, n = 5–17/time point) mice were inoculated with 10 µl of diluent and assessed for weight loss. Each data point represents the arithmetic mean ±SD of weights measured across twelve different experiments.
(TIF)

Figure S2 **MBL does not neutralize RRV.** To determine if RRV could be directly neutralized by complement components within serum, 10^3^ PFU of RR64 was incubated with increasing amounts of naïve mouse serum from wild-type (solid circle) or MBL-DKO mice (solid square), or heat-inactivated wild-type serum (open circle) for 1 hour at 37°C. The number of plaques was determined by plaque assay on BHK-21 cells. Each data point represents the arithmetic mean ± SD of three replicates and is representative of three independent experiments.
(TIF)

Figure S3 **Complement activation within quadriceps muscle is largely dependent on MBL.** (A–B). (A) To determine if complement activation was dependent on MBL, we analyzed homogenized quadriceps muscles from either mock- or RRV-infected WT or MBL-DKO mice 7 dpi by immunoblot analysis using an anti-mouse C3 or anti-mouse actin antibody. C3 cleavage products are indicated with solid arrowheads. Each lane represents an individual mouse, and the western blot is representative of three independent experiments (B) Densitometry measurements of bands in immunoblots from three independent experiments are graphically depicted as arbitrary units normalized to actin (RRV-infected WT mice, solid circle n = 9; MBL-DKO mice, open circle n = 10) (C) To determine if either the classical or alternative complement activation pathways contribute to C3 deposition following RRV infection, we performed IHC using an anti-mouse C3 on quadriceps muscle sections from RRV-infected wild-type, C1q^−/−^, or fB^−/−^ at 10 dpi. C3 deposition is shown in red for C1q^−/−^ and the wild-type control, and in blue for the fB^−/−^ and wild-type control. 5 µm thick paraffin-embedded sections were prepared as described in the Materials and Methods, with the following modification: RRV-infected C1q^−/−^ and wild-type sections were developed using Vector Red Alkaline phosphatase substrate kit (Vector Labs, CA) instead of Vector Blue Alkaline phosphatase substrate kit. A representative section from each strain is shown (n = 3 for wild-type mice, n = 3 for C1q^−/−^ mice; n = 3 for wild-type mice, n = 3 for fB^−/−^ mice).
(TIF)

Figure S4 **Inflammatory cell composition is not significantly different in RRV infected WT and MBL-DKO mice.** (A–B) Leukocytes were isolated from the quadriceps muscle of RRV-infected WT mice (solid circle, n = 5–12/time point) or MBL-DKO (open circle, n = 7–12/time point) at 7 or 10 dpi. Cells were characterized and quantified by flow cytometry using the markers described in the Materials and Methods. Total numbers of leukocytes (A) and specific cells types (B) are shown. Each data point represents an individual mouse and data presented in this figure are combined from three independent experiments (7 dpi). *p<0.05 by Mann-Whitney analysis.
(TIF)

Figure S5 **Representative flow cytometry plots and gating scheme used to characterize inflammatory infiltrates.** (A) To determine the number of leukocytes, we gated on LCA^+^ cells. To further distinguish between NK cells and T cells, we analyzed expression of NK1.1 and CD3 on LCA^+^ lymphocytes. NK1.1^+^CD3^−^ were classified as NK cells, and NK1.1^−^CD3^+^ cells were classified as T cells. T cells were further classified into CD4^+^ and CD8^+^ T cells based on CD4 and CD8 expression. Percentages displayed on plots represent the percentage of cells within the indicated gate. (B) To determine the number of inflammatory macrophage, we first gated on LCA^+^ cells, followed by analysis of CD11b and B220 expression. Inflammatory macrophage typically stain CD11b^+^B220^−^. We distinguished inflammatory macrophage from neutrophils within the CD11b^+^B220^−^ population by analyzing F4/80 and Gr-1/Ly-6G expression; inflammatory macrophage were defined as CD11b^+^B220^−^F4/80^+^Gr-1^lo^. Percentages displayed on plots represent the percentage of cells within the indicated gate.
(TIF)
