# Supplementary figures and images for: Mannose Binding Lectin Is Required for Alphavirus-Induced Arthritis/Myositis
Source: PLoS Pathog. 2012 Mar 22;8(3):e1002586. doi: 10.1371/journal.ppat.1002586 (PMC3310795; doi:10.1371/journal.ppat.1002586)

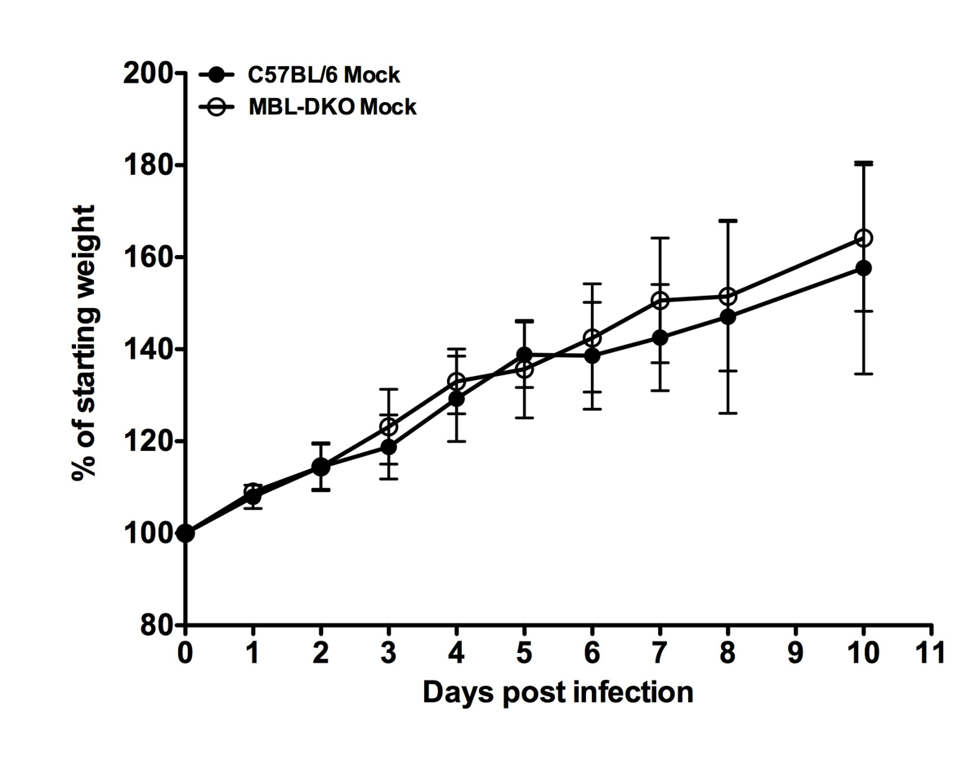

Supplement: Figure S1 — C57BL/6 wild-type and MBL-DKO mock-infected control mice exhibit weight gain throughout course of experiments. Twenty-four day old WT C57BL/6 (solid circle, n = 5–13/time point) or MBL-DKO (open circle, n = 5–17/time point) mice were inoculated with 10 µl of diluent and assessed for weight loss. Each data point represents the arithmetic mean ±SD of weights measured across twelve different experiments. (TIF) [file ppat.1002586.s001.tif]

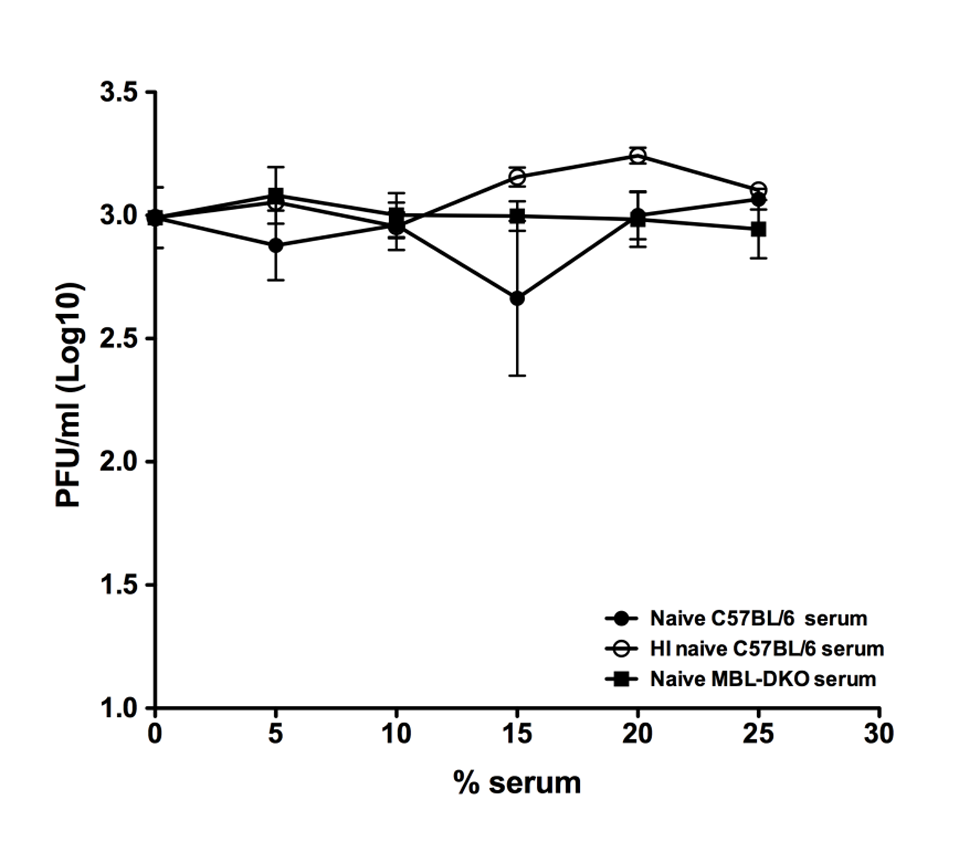

Supplement: Figure S2 — MBL does not neutralize RRV. To determine if RRV could be directly neutralized by complement components within serum, 103 PFU of RR64 was incubated with increasing amounts of naïve mouse serum from wild-type (solid circle) or MBL-DKO mice (solid square), or heat-inactivated wild-type serum (open circle) for 1 hour at 37°C. The number of plaques was determined by plaque assay on BHK-21 cells. Each data point represents the arithmetic mean ± SD of three replicates and is representative of three independent experiments. (TIF) [file ppat.1002586.s002.tif]

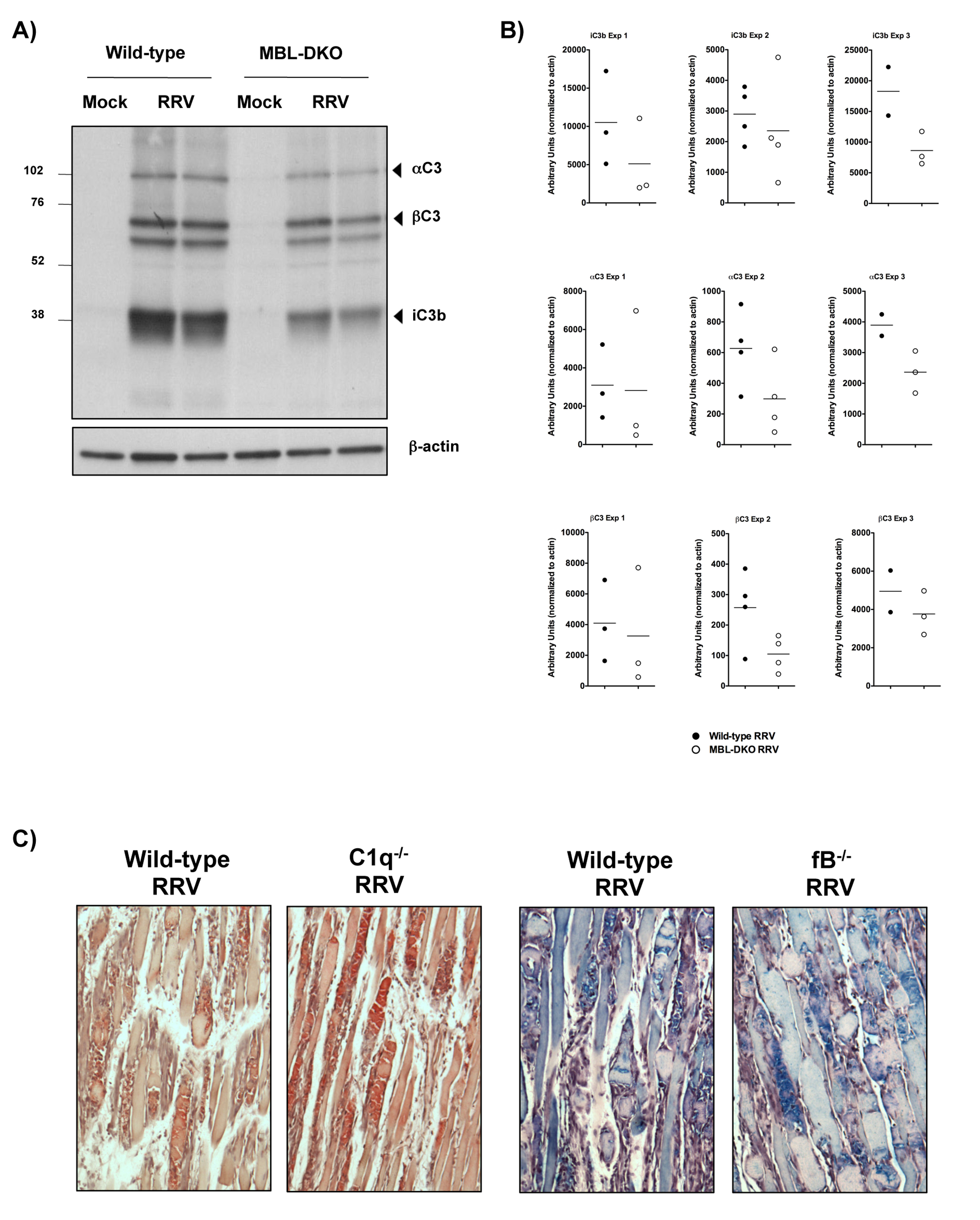

Supplement: Figure S3 — Complement activation within quadriceps muscle is largely dependent on MBL. (A–B). (A) To determine if complement activation was dependent on MBL, we analyzed homogenized quadriceps muscles from either mock- or RRV-infected WT or MBL-DKO mice 7 dpi by immunoblot analysis using an anti-mouse C3 or anti-mouse actin antibody. C3 cleavage products are indicated with solid arrowheads. Each lane represents an individual mouse, and the western blot is representative of three independent experiments (B) Densitometry measurements of bands in immunoblots from three independent experiments are graphically depicted as arbitrary units normalized to actin (RRV-infected WT mice, solid circle n = 9; MBL-DKO mice, open circle n = 10) (C) To determine if either the classical or alternative complement activation pathways contribute to C3 deposition following RRV infection, we performed IHC using an anti-mouse C3 on quadriceps muscle sections from RRV-infected wild-type, C1q−/−, or fB−/− at 10 dpi. C3 deposition is shown in red for C1q−/− and the wild-type control, and in blue for the fB−/− and wild-type control. 5 µm thick paraffin-embedded sections were prepared as described in the Materials and Methods, with the following modification: RRV-infected C1q−/− and wild-type sections were developed using Vector Red Alkaline phosphatase substrate kit (Vector Labs, CA) instead of Vector Blue Alkaline phosphatase substrate kit. A representative section from each strain is shown (n = 3 for wild-type mice, n = 3 for C1q−/− mice; n = 3 for wild-type mice, n = 3 for fB−/− mice). (TIF) [file ppat.1002586.s003.tif]

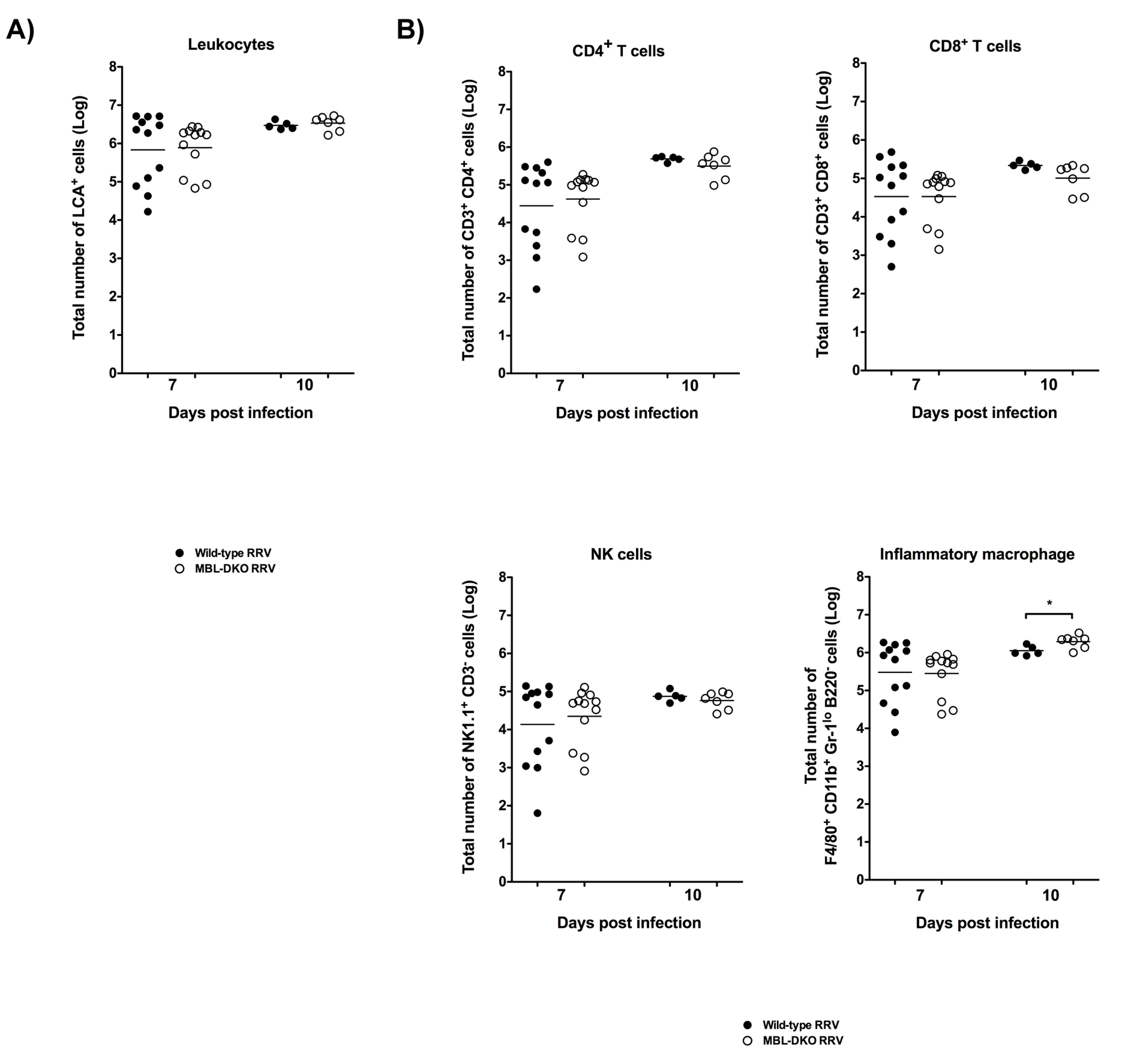

Supplement: Figure S4 — Inflammatory cell composition is not significantly different in RRV infected WT and MBL-DKO mice. (A–B) Leukocytes were isolated from the quadriceps muscle of RRV-infected WT mice (solid circle, n = 5–12/time point) or MBL-DKO (open circle, n = 7–12/time point) at 7 or 10 dpi. Cells were characterized and quantified by flow cytometry using the markers described in the Materials and Methods. Total numbers of leukocytes (A) and specific cells types (B) are shown. Each data point represents an individual mouse and data presented in this figure are combined from three independent experiments (7 dpi). *p<0.05 by Mann-Whitney analysis. (TIF) [file ppat.1002586.s004.tif]

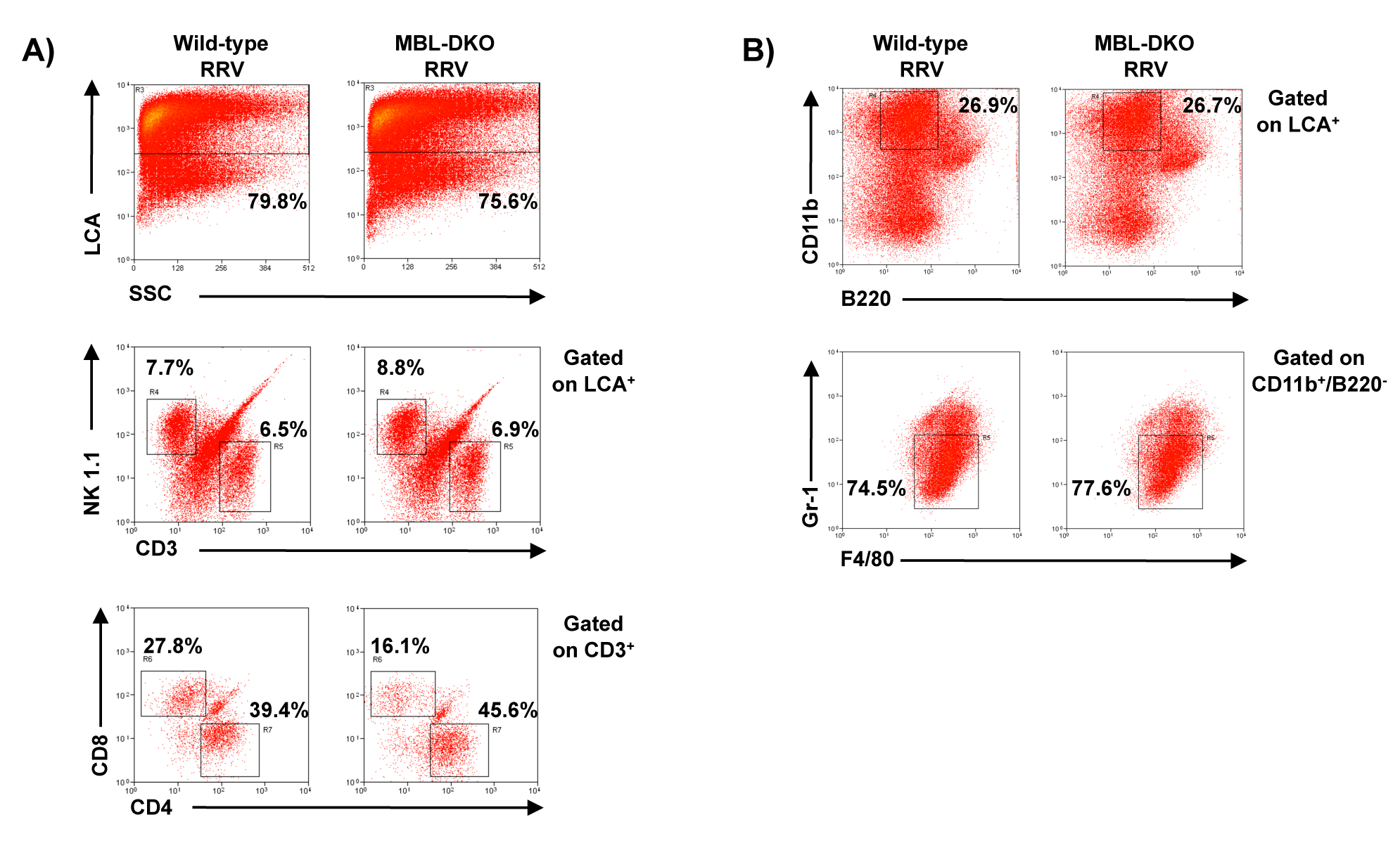

Supplement: Figure S5 — Representative flow cytometry plots and gating scheme used to characterize inflammatory infiltrates. (A) To determine the number of leukocytes, we gated on LCA+ cells. To further distinguish between NK cells and T cells, we analyzed expression of NK1.1 and CD3 on LCA+ lymphocytes. NK1.1+CD3− were classified as NK cells, and NK1.1−CD3+ cells were classified as T cells. T cells were further classified into CD4+ and CD8+ T cells based on CD4 and CD8 expression. Percentages displayed on plots represent the percentage of cells within the indicated gate. (B) To determine the number of inflammatory macrophage, we first gated on LCA+ cells, followed by analysis of CD11b and B220 expression. Inflammatory macrophage typically stain CD11b+B220−. We distinguished inflammatory macrophage from neutrophils within the CD11b+B220− population by analyzing F4/80 and Gr-1/Ly-6G expression; inflammatory macrophage were defined as CD11b+B220−F4/80+Gr-1lo. Percentages displayed on plots represent the percentage of cells within the indicated gate. (TIF) [file ppat.1002586.s005.tif]
